# Supplementary material for: Anomalous Perception of Biological Motion in Autism: A Conceptual Review and Meta-Analysis
Source: Sci Rep. 2020 Mar 12;10:4576. doi: 10.1038/s41598-020-61252-3 (PMC7067769; doi:10.1038/s41598-020-61252-3)
Supplement: Supplementary file 1 — Supplementary information. [file 41598_2020_61252_MOESM1_ESM.pdf]

Supplementary information to the article:

ANOMALOUS PERCEPTION OF BIOLOGICAL MOTION IN AUTISM: A CONCEPTUAL REVIEW AND META-ANALYSIS

Alessandra Federici[1,2§], Valentina Parma[3,4,§], Michele Vicovaro[5], Luca Radassao[3],

Luca Casartelli[1§§*], Luca Ronconi[1,6,7§§*]

[1] Child Psychopathology Unit, Theoretical and Cognitive Neuroscience Group, Scientific Institute IRCCS E. Medea, Bosisio Parini, Lecco, Italy
[2] MoMiLab Research Unit, IMT School of Advanced Studies Lucca, Lucca, Italy
[3] International School for Advanced Studies (SISSA), Trieste, Italy
[4] Department of Psychology, Temple University, Philadelphia, PA, United States
[5] Department of General Psychology, University of Padova, Padova, Italy
[6] School of Psychology, Vita-Salute San Raffaele University, Milan, Italy
[7] Division of Neuroscience, IRCCS San Raffaele Scientific Institute, Milan, Italy

§ = co-first authorship

§§= co-last authorship

Figure S1. Number of articles published per year as indexed in PubMed with the general search using the keywords “biological motion autism”.

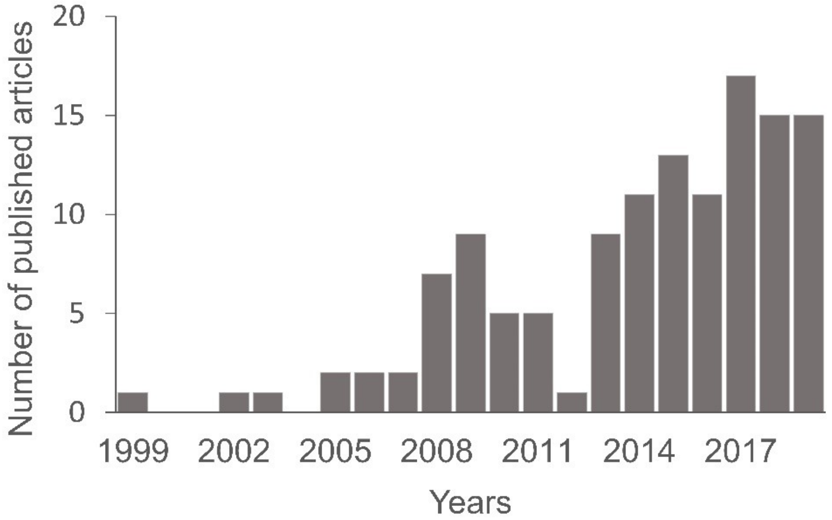

Table S1. The table contains the final set of studies included in the meta-analysis. For each study descriptive statistics are reported for both ASD and TD groups (since different tests were used in order to assess the participants' cognitive level, in the subcolumn 'Test' the name of the test is specified). When the data were not available in the paper N.A.is reported. The column 'Meta-analysis and related categorization' contains four subcolumns where we reported in which meta-analysis each study was included (1=All BM studies, 2=Level of processing, 3=Low-level features, 4=Non-BM), and the relative categorization: Level of processing (First-order, Direct, Instrumental), Type of scrambled (Spatial, Temporal, Spatial and Temporal) and Type of stimuli (BM=only biological motion stimuli, non-BM=only non-Biological motion stimuli, BM vs. non-BM when a biological stimulus has to be discriminated from a non-biological one). In the 'Task characteristics' column we reported which behavioral measure was assessed in the task (Accuracy, % Looking), if the task was performed during an EEG or a fMRI acquisition, and a brief description of the implemented task.

STUDY	GROUP		% MALE		AGE Mean±SD		COGNITIVE LEVEL Mean±SD			META-ANALYSIS AND RELATED CATEGORIZATION				TASK CHARACTERISTICS		
	ASD	TD	ASD	TD	ASD	TD	Test	ASD	TD	Meta-analysis performed	Level of Processing	Type of scrambled	Type of stimuli	Behavioral measure	Neuro acquisition	Qualitative description of task
Blake et al., 2003	12	9	100%	100%	range 8-10	8.42 ±1.89	Kaufman Assessment Battery for Children, or K-ABC	7.9 ±1.8	N.A.	1, 2, 3	Direct	Temporal	BM vs. non-BM	Accuracy	/	Twenty-five human actions (e.g., running, kicking, climbing, throwing, jumping) and their scrambled version were presented. Each participant verbally reported whether the sequence was "a person" or "not a person."
Herrington et al., 2007	10	10	100%	100%	27.60 ±7.10	25.6 ±4.9	Wechsler Abbreviated Scale of Intelligence	109 ±N.A.	119 ±N.A.	1, 2, 3	Direct	Spatial	BM	Accuracy	fMRI	Participants indicated with pressing the right or left botton whether a PLD human image or its scrambled version is walking either to the left or to the right side of the screen.
										4	/	Spatial	non-BM	Accuracy	fMRI	
Hubert et al., 2007	19	19	89%	89%	21.5 ±6.08	24.3 ±8.5	Wechsler Scale (WAIS or WISC)	83.3 ±15.9	N.A	1, 2	Instrumental	/	BM	Accuracy	/	Participants were asked to describe orally what is happening in a series of movies that shows 10 common actions , 5 subjective states, 5 emotional states, and 5 objects.
										4	/	/	non-BM	Accuracy	/	
Freitag et al., 2008	13	15	87%	87%	17.6 ±3.6	18.6 ±1.2	Wechsler scale	101.2 ±21.1	112.1 ±N.A.	1, 2	Direct	Spatial and Temporal	BM vs. non-BM	Accuracy	/	Participants were asked to press different buttons if they see either a walking person (40 famele and 40 male) or a scrambled version.
Atkinson, 2009	13	16	92%	88%	30.9 ±13.8	26.7 ±12.8	ASD Wechsler Scale. TD IQ derived by the revised version of the National Adult Reading Test	106.2 ±12.2	106.6 ±8.5	1, 2	Instrumental	/	BM	Accuracy	/	Participants were asked to indicate which emotion label, among 5 emotions (anger, disgust, fear, happiness and sadness), or which action label, among 8 human actions (digging, kicking, knocking, pushing, bending to touch toes, hopping,walking on the spot, and star-jumping or jumping jacks), best described the displayed movie clip.

Klin et al., 2009	21	39	100%	100%	2.21 ±0.54	1.99 ±0.66	Visual Reception subtest of the Mullen Scales of Early Learning	2 ±0.95	2.1 ±0.91	1, 2	First-order	/	BM	% Looking	/	In a preferential looking paradigm an animations consisted of children's games (e.g., playing "peek-a-boo" or "pat-a-cake") was presented in half of the screen. On the other half of the screen, the same animation was presented inverted (shown upside-down) and played in reverse order. Only the one (forward) audio soundtrack was presented.
Murphy et al., 2009	16	16	81%	81%	25.56 ±7.67	26.40 ±2.8	Raven	43.73 ±9.64	56.21 ±N.A.	1, 2	Direct	Spatial and Temporal	BM	Accuracy	/	A human walker or its scrambled version was displayed in a noisy dots background. On each trial participants were asked to respond as quickly as possible with two differnet buttons whether the stimulus was moving to the left or the right.
										4	/	Spatial and Temporal	non-BM	Accuracy	/	
Annaz et al., 2010	23	34	100%	100%	8.83 ±1.83	8.25 ±2.25	British Picture Vocabulary Scale Pattern II	7.17 ±1.67	8.42 ±2.25	1, 2, 3	Direct	Temporal	BM vs. non-BM	Accuracy	/	In the first task a human action (i.e., walking, running, throwing, kicking and star-jumping) or its scrambled version was displayed; the participant has to identify if the stimulus was moving like a person or not. In the second task a walker human or its scrambled version were presented side-by-side in a noisy dots background; the participant has to select the panel (left or right) where the dots look like a person.
							Construction subtest from the british ability scale II	8.92 ±3.08	8.25 ±2.08							
Koldewyn et al., 2010	25	32	92%	94%	15.12 ±2.64	15.78 ±2.41	Wechsler Scale (WASI)	107.8 ±16	121.3 ±2.41	1, 2	Direct	/	BM	Accuracy	/	Participants were required to report in which direction the figure was walking (left or right). The walker was embaded in a mask that could have different levels of coherence.
Saygin et al., 2010	16	20	81%	65%	33.75 ±12.7	37.75 ±11.35	IQ	112.19 ±16.25	113.16 ±12.35	1, 2	Direct	/	BM	Accuracy	/	Participants were required to report in which direction the figure was walking (left or right). The walker was embaded in noise dots, each of them with the same trajectory of one of the dots in the walker.

Annaz et al., 2012	17	17	100%	100%	5.5 ±1.08	5.58 ±1.08	British Picture Vocabulary Scale Pattern II	5.17 ±1.08	6.58 ±1.17	1, 2, 3	First-order	Temporal	BM vs. non-BM	% Looking	/	In a preferential looking paradigm a human walking and its scrambled version were displayed side by side for 6 sec.
							Construction subtest from the british ability scale II	6.33 ±1.25	6.41 ±1.08	1	First-order	/	BM vs. non-BM	% Looking	/	In a preferential looking paradigm a human walking and a moving object (spinning top) were displayed side by side for 6 sec.
McKay et al., 2012	10	10	100%	100%	28.6 ±6.92	27.9 ±7.37	Wechsler Scale (WASI)	125 ±7.01	124.8 ±6.7	1, 2, 3	Direct	Spatial	BM vs. non-BM	Accuracy	/	A walker or its scrambled version was presented embaded in a number of noise dots. Participants were required to report in which direction the stimulus is moving (toward the left or the right).
Nackaerts et al., 2012	12	12	58%	58%	34.9 ±8.5	31.5 ±6.3	Wechsler Scale (Ward 7-subtest short-form)	111.5 ±18.2	115.5 ±15.1	1, 2, 3	Direct	Spatial	BM vs. non-BM	Accuracy	/	A human action (walking, jumping on the spot or kicking) with an emotional states (neutral, happy, sad or angry) or a scrambled version was displayed and the participant had to indicate as fast and accurate as possible whether the stimlus was ''a person'' or ''not a person'.
										1, 2	Instrumental	/	BM	Accuracy	/	A prime and then a target stimulus were shown. Participant had to judge if the target (a human walking, jumping on the spot or kicking who could be happy, sad, angry or neutral) showed the same emotion of the prime stimulus.
Price et al., 2012	14	16	100%	87.5%	14.14 ±4.8	14.08 ±4.61	Wechsler Scale (WASI)	60.14 ±9.44	57.81 ±9.29	1, 2	Direct	/	BM	Accuracy	/	The stimuli were 28 typical gaits half coherent - gait of one person, half incoherent leg movements from two different walkers and 27 depicting gaits with limps. Participants made two judgments of each PLD walker: judging whether the stimulus depicted a coherent or incoherent gait, and whether each walker depicted a normal gait or a limp.

Rutherford & Troje 2012	13	14	100%	100%	29±6	31±9	Wechsler Scale (WAIS)	98.1 ±13.61	94.6 ±10.03	1, 2, 3	Direct	Spatial	BM vs. non-BM	Accuracy	/	The stimuli represent human, cat or pigeon walking (upright or inverted) or their scrambled version, the stimulus was embedded into the scrambled-walker mask. Two tasks were performed: in the first, the subject had to select if the coherent walker was displayed in the first or in the second stimulus; in the second, the subject should judge the directions of the stimulus.
Falck-Ytter et al., 2013	10	14	80%	79%	3.42 ±0.44	3.55 ±0.43	Visual	1.89 ±0.64	3.69 ±0.93	1, 2	First-order	/	BM	% Looking	/	In a preferential looking paradigm a video of a human action (e.g., clapping hands) and its up-side down version were shown side by side on the screen.
							Language	1.44 ±0.63	3.55 ±0.6							
Alaerts et al., 2014 - 2017	15	15	100%	100%	21.7 ±4	23.3 ±2.9	Wechsler Scale	107.9 ±13.9	114.8 ±12.8	1, 2	Instrumental	/	BM	Accuracy	fMRI	Participants had to indicates if a human (walking, jumping or kicking) is happier, sadder, angrier or not different from the previous one; the first stimulus showed always a neutral emotional state.
	15	14								1, 2, 3	Direct	Spatial	BM vs. non-BM	Accuracy	fMRI	Participants were presented an intact BM (walking, jumping or kicking in four different 'emotional states' neutral, happy, sad, angry) or the scrambled version. They have to indicate as fast and accurate as possible whether the presented PLD represented 'a person' or 'not a person'.
Kröger et al., 2014	17	21	100%	100%	11.9 ±2.2	11.63 ±2.4	Raven	77.6 ±30.2	59.1 ±38.2	1, 2, 3	Direct	Spatial	BM	Accuracy	EEG	30 different walkers and 30 scrambled motion stimuli were shown. Participants had to indicate if they have seen a walker or just a motion pattern.
										4	/	Spatial	non-BM	Accuracy	EEG	
Wright et al., 2014	19	21	84%	62%	6.96 ±1.5	6.49 ±1.77	Non verbal mental age	103.14 ±14.59	7.3 ±2.84	1, 2	Direct	/	BM	Accuracy	/	After the presentation of the moving stimulus (i.e., a human, a cat, a truck or a bicycle), the children have to verbally answer at the question "What is that?"
										4	/	/	non-BM	Accuracy	/	

Cusack et al., 2015	15	15	100%	100%	16.09 ±2.24	15.54 ±2.15	Wechsler Abbreviated Scale	103.14 ±11.59	104.79 ±9.14	1, 2	Instrumental	/	BM	Accuracy	/	Participant have to discriminate if two agents were fighting or dancing in a normal or upside-down condition
										1, 2, 3	Direct	Temporal	BM vs. non-BM	Accuracy	/	1.Participants were asked to discriminate between a BM and its scrambled version. 2. Participants were asked to select the BM, in which limbs were intact vs. its version with scrambled limbs. Both tasks have an upright and inverted conditions.
Wang et al., 2015	21	21	81%	76%	67.05 ±15.21	59.38 ±16.99	verbal score Language test	28.05 ±30.27	60.11 ±24.83	1, 2, 3	First-order	Spatial	BM vs. non-BM	% Looking	/	In a preferential looking paradigm each trial present a human or a pigion walking and its scrambled version side by side for 10 sec.
										1, 2	Instrumental	/	BM	Accuracy	/	In this task the subject saw a short movie where one of 12 different human actions (e.g., standing up, sitting down, walking, running) was presented. Participants had to verbal respond as soon as they have indentifed the action displayed.
Fujioka et al., 2016	21	35	100%	100%	27.6 ±7.7	25.2 ±4.5	Wechsler Scale (WAIS-WISC)	99.8 ±13.5	N.A.	1, 2	First-order	/	BM	% Looking	/	In a preferential looking paradigm a movie presents upright and inverted BM simultaneously side by side for 20 sec.
von der Lühe et al., 2016	16	16	75%	62.5%	41.56 ±9.15	36.19 ±12.11	WST (Wortschatz test, German multiple-choice vocabulary test)	116.88 ±15.59	115.31 ±8.43	1, 2, 3	Direct	Temporal	BM vs. non-BM	Accuracy	/	The stimlus was composed by two intervals both containing an agent A, and only one of them containing the agent B; the other interval contained a scrambled version of the agent B. Agent B or its scrambled version was masked by dots. Participants had to decided which interval contains the agent B.
										1, 2	Instrumental	/	BM	Accuracy	/	Participants performed an explicit intention recognition task on 21 videos depicting two PLD agents (14 comunicative and 7 indipendent actions).

Wright et al., 2016	18	18	83%	83%	6.61 ±1.29	6.41 ±1.75	verbal IQ	102 ±19.3	105.22 ±11.44	1, 2	First-order	/	BM vs. non-BM	% Looking	/	In a preferential looking paradigm participants watched a video with one animate (i.e., human or cat) and one inanimate (i.e., trucks or bicycle) PLD moving side by side on the screen.
							non verbal IQ	102.82 ±16.7	106.61 ±9.88							
Burnside et al., 2017	15	16	100%	50%	5.22 ±1.36	3.98 ±1.49	DAS - Non Verbal mental	4.36 ±1.87	4.28 ±1.56	1, 2, 3	First-order	Temporal	BM vs. non-BM	% Looking	/	In a preferential looking paradigm a walking human and its scrambled version were presented side by side on the screen for 6 sec.
							PPTV verbal metal age	4.06 ±1.85	4.72 ±1.79							
Turi et al., 2017	19	18	84.21%	77.78%	11.49 ±2.24	11.94 ±2.73	Wechsler Scale (WASI)	105.9 ±16.68	107.6 ±9.21	1, 2	Instrumental	/	BM	Accuracy	/	Subjects observed a movies of a hand grasping an invisible object, and had to guess whether the goal of the movement was towards a small cylinder or large cube. The moviment could be presented with an egocentrinc or an allocentric view.
Lindor et al., 2019	18	13	53.8%	61.25%	9.69 ± 1.85	9.42 ± 2.64	WISC-IV	101.5 ± 10.09	111.69 ± 15.22	1, 2	Instrumental	No	BM	Accuracy	/	Participants observed 6 different of communicative interactions performed by two PLD actors (duration between 3.5 and 7.5 sec). Each interaction was played twice per trial before participants were asked to verbally explain "what was going on in the video." Each video was presented half time upright trials and the other half inverted.
Hsiung et al., 2019	27	30	55.56%	50,00%	28.4 ± 5.46	22.4 ± 1.86	Educational level	15.6 ± 1.69	15.8 ± 0.70	1, 2	Instrumental	No	BM	Accuracy	/	Twenty PLD different actions (eg.walking or playing tennis) were pesented. In each trial, an action was played three times. The participant was asked to press the space bar as quickly as possible once they recognised the action and verbally answer to the question 'what is the action?'

Sotoodeh et al., 2019	20	20	86.36%	8.%	11.3 ± 2.1	11.4 ± 2.8	IQ (non-verbal)	77.3 ± 4.93	106 ± 12.86	1, 2	Instrumental	No	BM	Accuracy	EEG	Paticipants performed an action identification task. In each trial one of the 4 possible PLD actions (i.e., walking, under-arm throwing, basketball free throwing and dancing) was displayed 3 times. Children were asked to recognise the presented PLD and respond as fast and accurately as possible

References

Blake, R., Turner, L. M., Smoski, M. J., Pozdol, S. L. & Stone, W. L. Visual recognition of biological motion is impaired in children with autism. Psychol Sci 14, 151–157 (2003).

Klin, A., Lin, D. J., Gorrindo, P., Ramsay, G. & Jones, W. Two-year-olds with autism orient to non-social contingencies rather than biological motion. Nature 459, 257–261 (2009).

Saygin, A. P., Cook, J. & Blakemore, S. J. Unaffected perceptual thresholds for biological and non-biological form-from-motion perception in autism spectrum conditions. PLoS One 5, 1–7 (2010).

Annaz, D., Campbell, R., Coleman, M., Milne, E. & Swettenham, J. Young children with autism spectrum disorder do not preferentially attend to biological motion. J. Autism Dev. Disord. 42, 401–408 (2012).

Price, K. J., Shiffrar, M. & Kerns, K. A. Movement perception and movement production in Asperger's Syndrome. Res. Autism Spectr. Disord. 6, 391–398 (2012).

Falck-Ytter, T., Rehnberg, E. & Bölte, S. Lack of Visual Orienting to Biological Motion and Audiovisual Synchrony in 3-Year-Olds with Autism. PLoS One 8, 3–7 (2013).

Alaerts, K. et al. Underconnectivity of the superior temporal sulcus predicts emotion recognition deficits in autism. Soc. Cogn. Affect. Neurosci. 9, 1589–1600 (2014).

Cusack, J. P., Williams, J. H. G. & Neri, P. Action Perception Is Intact in Autism Spectrum Disorder. J. Neurosci. 35, 1849–1857 (2015).

Wang, L. H., Chien, S. H. L., Hu, S. F., Chen, T. Y. & Chen, H. S. Children with autism spectrum disorders are less proficient in action identification and lacking a preference for upright point-light biological motion displays. Res. Autism Spectr. Disord. 11, 63–76 (2015).

Fujioka, T. et al. Gazefinder as a clinical supplementary tool for discriminating between autism spectrum disorder and typical development in male adolescents and adults. Mol. Autism 7, 19 (2016).

von der Luhe, T. et al. Interpersonal predictive coding, not action perception, is impaired in autism. Philos. Trans. R. Soc. B Biol. Sci. 371, 20150373 (2016).

Burnside, K., Wright, K. & Poulin-Dubois, D. Social motivation and implicit theory of mind in children with autism spectrum disorder. Autism Res. 1–11 (2017). doi:10.1002/aur.1836

Turi, M., Muratori, F., Tinelli, F., Morrone, M. C. & Burr, D. C. Autism is associated with reduced ability to interpret grasping actions of others. Sci. Rep. 7, 1–8 (2017).

Lindor, E. R., van Boxtel, J. J. A., Rinehart, N. J. & Fielding, J. Motor difficulties are associated with impaired perception of interactive human movement in autism spectrum disorder: A pilot study. J. Clin. Exp. Neuropsychol. 41, 856–874 (2019).

Hsiung, E. Y., Chien, S. H. L., Chu, Y. H. & Ho, M. W. R. Adults with autism are less proficient in identifying biological motion actions portrayed with point-light displays. J. Intellect. Disabil. Res. 63, 1111–1124 (2019).

Sotoodeh, M. S., Taheri-Torbati, H., Sohrabi, M. & Ghoshuni, M. Perception of biological motions is preserved in people with autism spectrum disorder: electrophysiological and behavioural evidences. J. Intellect. Disabil. Res. 63, 72–84 (2019).
